# Gastrointestinal Cancers in Hospitalized Patients with Cystic Fibrosis: A Nationwide Study, 2010–2020

**DOI:** 10.3390/diagnostics14181999

**Published:** 2024-09-10

**Authors:** Paul Wasuwanich, Wikrom Karnsakul

**Affiliations:** 1 Department of Internal Medicine, Naples Comprehensive Health, Naples, FL 34102, USA; 2 Department of Internal Medicine, University of Florida College of Medicine, Gainesville, FL 32610, USA; 3 Division of Pediatric Gastroenterology, Hepatology, and Nutrition, Department of Pediatrics, Johns Hopkins University School of Medicine, 550 N. Broadway, 10th Floor Suite 1003, Baltimore, MD 21205, USA

**Keywords:** cystic fibrosis, pancreatic cancer, liver cancer, small bowel cancer, colorectal cancer

## Abstract

Background: As life expectancy in cystic fibrosis (CF) patients has increased, so has the incidence of cancers. We aimed to investigate and describe gastrointestinal cancers in CF hospitalized patients from 2010 to 2020. Methods: Utilizing the National Inpatient Sample, we extracted cases of CF-associated hospitalizations and gastrointestinal cancers as well as demographic and clinical data. We compared our CF cohort to age, sex, and race/ethnicity-matched controls. Trends were analyzed by Poisson regression. Results: We identified a total of 902 hospitalizations of CF with gastrointestinal cancer; among them, 539 (59.8%) were colorectal, 139 (15.4%) were liver, 105 (11.6%) were pancreatic, 54 (6.0%) were small bowel, 35 (3.9%) were gastric, and 30 (3.3%) were esophageal cancers. The median age of hospitalization for gastrointestinal cancers ranged from 39 years in liver cancer to 65 years in small bowel cancer. Mortality ranged from 9.5% in pancreatic to 0.0% in small bowel cancer. Colorectal cancer (IRR: 1.09; *p* = 0.005), pancreatic cancer (IRR: 1.17; *p* = 0.023), gastric cancer (IRR: 1.41; *p* = 0.003), and esophageal cancer (IRR: 1.50; *p* = 0.023) hospitalization rates have been increasing over time. Rates of colorectal (*p* = 0.037) cancer were significantly higher in our CF cohort compared to controls. Conclusions: Colorectal cancers are the major gastrointestinal cancers in CF patients, and the incidence of these hospitalizations is increasing.

## 1. Introduction

Cystic fibrosis (CF) has historically been considered the most common life-threatening genetic disorder among populations of European descent, with an estimated incidence of 1/3000 to 1/6000 live births in that population [1,2]. CF is caused by a mutation in the cystic fibrosis transmembrane conductance regulator (CFTR) gene resulting in dysfunction or absence of the corresponding CFTR protein.

With improvements in therapy, life expectancy in the CF population has been improving over time. The median age of death for an individual living with CF was 33.9 years in 2021, and the median predicted age of survival for an individual born with CF in 2021 was 53.1 years [3]. The primary cause of mortality in the CF population continues to be respiratory/cardiorespiratory, attributing to 44.4% of deaths [3]. However, as life expectancy continues to increase each year, so does the burden of cancer.

As early as the 1990s, cancer risks in the CF population were being investigated. An early study by Neglia et al. in 1995 reported that patients with CF had similar risks of developing cancer overall compared to the general population, but also found that for gastrointestinal cancers specifically, there was a notable increased risk in the CF population [4]. A more recent study by Maisonneuve et al. in 2013 also found that overall cancer risk in CF patients was similar to the general population, but only in CF patients who were not solid organ transplant recipients [5]. However, in CF patients who received solid organ transplants, Maisonneuve et al. reported that overall cancer was increased compared to the general population [5]. The group also reported increased risks of gastrointestinal cancers, testicular cancer, and lymphoid leukemia in the CF population [5]. It is important to note, however, that patients who receive solid organ transplants are generally at increased risk of developing cancer and this is not unique to the CF population [6]. A systematic review and meta-analysis in 2018 by Yamada et al. reported significantly increased risk of small bowel cancer, colon cancer, biliary tract cancer, and pancreatic cancer with pooled standardized incidence ratios of 18.94, 10.91, 17.87, and 6.18, respectively, compared to the general population. The authors estimated a pooled incidence rate of gastrointestinal cancers in the CF population to be 0.79 per 1000 person-years. Additionally, they estimated the incidence rates for small bowel cancer, colon cancer, biliary tract cancer, and pancreatic cancer to be 0.13, 0.39, 0.051, and 0.058 per 1000 person-years, respectively [7].

We aim to describe the demographic and clinical characteristics of hospitalized CF patients with gastrointestinal cancers and compare the rates of those cancers to age-, sex-, and race/ethnicity-matched hospitalized non-CF patients with asthma. 

## 2. Materials and Methods

### 2.1. Study Population

After receiving approval from the institutional review board of the Johns Hopkins University School of Medicine, we utilized data from the National Inpatient Sample, a database on inpatient stays and hospital discharges in the United States from the Healthcare Cost and Utilization Project (HCUP) [8]. Compared to the Cystic Fibrosis Foundation registry data, the National Inpatient Sample provides more specific data on types of cancer and is available over several years. Data from 1 January 2010 to 31 December 2020 (the most recent year for which data were available) were extracted and analyzed. 

To obtain our primary study cohort, we included all hospitalizations with a CF diagnosis that occurred between 1 January 2010 and 31 December 2020 and had a concurrent diagnosis of one of the following six gastrointestinal cancers of interest: colorectal cancer, liver cancer (including hepatocellular carcinoma and intrahepatic cholangiocarcinoma), pancreatic cancer, small bowel cancer, gastric cancer, and esophageal cancer. There were no exclusions based on age, sex, or race/ethnicity. 

The CF diagnosis was defined by the International Classification of Diseases, Ninth Revision (ICD-9) diagnosis code 277.0* and by the International Classification of Diseases, Tenth Revision (ICD-10) diagnosis code E84*. Hospitalizations with asthma, defined by ICD-9 diagnosis code 493* and ICD-10 diagnosis code J45*, without a concurrent diagnosis of CF, were extracted and used as controls. Asthma is a primary respiratory disease that affects many children and young adults and can lead to hospitalization, but it is not associated with any increased risk of cancer, thus serving as a relatively ideal control population for CF cases. To account for the unique demographics of the CF population, CF cases were age-matched, sex-matched, and race/ethnicity-matched to computer-randomized asthma controls in a ratio of approximately 1 case to 2 controls, matching to age within ±1 year of cases and similar sex and race/ethnicity wherever possible.

We extracted demographic and geographic data including age, sex, race/ethnicity, region of hospital, and type of hospital (rural, urban non-teaching, or urban teaching). The following clinical data were obtained: length of hospital stay, all-cause mortality, colorectal cancer, liver cancer, pancreatic cancer, small bowel cancer, gastric cancer, esophageal cancer, history of solid organ transplantation, hepatitis B, and hepatitis C. The ICD-9 and ICD-10 diagnostic codes used for variables are presented in Supplementary Table S1.

### 2.2. Statistical Analysis

Hospitalization rates were analyzed for trends in the 2010 to 2020 period using Poisson regression and reported as incidence rate ratios (IRRs) per year. The denominator used in the calculation of hospitalization rates was the total United States population residing within the country on July 1st of the relevant year. Population data were extracted from the United States Census Bureau’s Population Estimates database [9]. Additionally, we also performed the Poisson regression using total CF hospitalizations from the National Inpatient Sample as the denominator for comparison. Quantitative data were tested for normality using the Kolmogorov–Smirnov test. The non-normal data were summarized using the median and interquartile range (IQR) and compared using the Mann–Whitney U test. Normally distributed data were analyzed with Student’s *t*-test. Frequencies were compared using the chi-squared test. Mortality rates were calculated using all-cause mortality within a specific group as the numerator and the entire population of that specific group as the denominator; mortality rates were reported in percentages. 

Results for categories that contain 10 or fewer hospitalizations but greater than zero hospitalizations were reported as ≤10 due to the data use privacy policy of HCUP. All reported *p*-values were two-tailed *p*-values. Statistical significance was defined as *p* < 0.05. Statistical calculations were performed using R program (R Core Team (2020). R: A language and environment for statistical computing. R Foundation for Statistical Computing, Vienna, Austria. URL https://www.R-project.org/ (accessed on 1 March 2024)).

## 3. Results

From 2010 to 2020, we identified a total of 323,594 hospitalizations with a diagnosis of CF. Among these, we identified 539 hospitalized with a concurrent diagnosis of colorectal cancer, 139 with liver cancer, 105 with pancreatic cancer, 54 with small bowel cancer, 35 with gastric cancer, and 30 with esophageal cancer.

**Figure 1:**
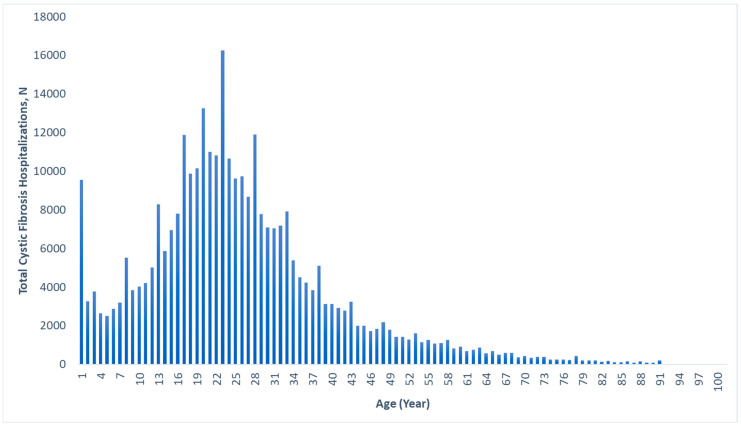
Age distribution of all cystic fibrosis-associated hospitalizations, 2010–2020.

The median age for hospitalization for CF in general was 22 (IQR: 16–32) years, with a range of 0–92 years. Figure 1 displays the age distribution of all CF hospitalizations, with a large percentage occurring in the 18–25 age range, and few after age 60. The median ages for hospitalization with gastrointestinal cancers within the CF cohort were 46 (IQR: 37–57) years for colorectal cancer, 39 (IQR: 21–54) years for liver cancer, 53 (IQR: 40–60) years for pancreatic cancer, 53 (IQR: 37–62) years for small bowel cancer, 65 (IQR: 54–79) years for gastric cancer, and 59 (IQR: 35–75) years for esophageal cancer (Table 1).

Among CF hospitalizations that involved colorectal cancer, female sex (51.0%) and non-Hispanic White race/ethnicity (82.7%) were most common. Most of these colorectal cancer hospitalizations occurred in the Southern region of the United States (38.6%) and at urban teaching hospitals (87.4%). Similarly, non-Hispanic White race/ethnicity was the most common demographic for the other gastrointestinal cancers as well. However, unlike with colorectal cancer, the other gastrointestinal cancers were predominantly male in demographics. The Southern region of the United States had the majority of hospitalizations for CF with liver cancer, pancreatic cancer, and esophageal cancer. Small bowel cancer in the CF cohort occurred predominately in the Midwestern region, and gastric cancer occurred predominately in the Northeastern region of the United States. By far, all CF hospitalizations with gastrointestinal cancers occurred in urban teaching hospitals (Table 1).

The mortality rates for CF hospitalizations with gastrointestinal cancers were 3.7% for colorectal, 7.2% for liver, 9.5% for pancreatic, 0.0% for small bowel, 28.6% for gastric, and 0.0% for esophageal cancer. In comparison, the mortality rates for our matched asthma controls with gastrointestinal cancers were 0.6% for colorectal, 2.6% for liver, 2.4% for pancreatic, 0.0% for small bowel, 1.8% for gastric, and 5.9% for esophageal cancer. Upon further investigation of the mortalities within the CF hospitalizations with gastrointestinal cancers, among the cancer groups where there were deaths, at least half had a concurrent diagnosis of either sepsis or pneumonia, with the exception of pancreatic cancer, where none of the patients who died had any concurrent diagnosis of sepsis or pneumonia.

The percentages of solid organ transplantation within the cohort of CF hospitalizations with gastrointestinal cancers were 19.3% for colorectal, 24.5% for liver, 0.0% for pancreatic, 35.2% for small bowel, 28.6% for gastric, and 50.0% for esophageal cancer. Further analysis of the CF liver cancer cohort revealed no cases of hepatitis B and ≤10 cases of hepatitis C.

The trends for general CF hospitalizations from 2010 to 2020 are displayed in Supplementary Figure S1. Overall, there was a decrease in the CF hospitalization rate over the 11-year period (IRR = 0.96; 95% CI = 0.96–0.96; *p* < 0.001) and a notable sharp drop in CF hospitalization rate in the year 2020.

**Figure 2:**
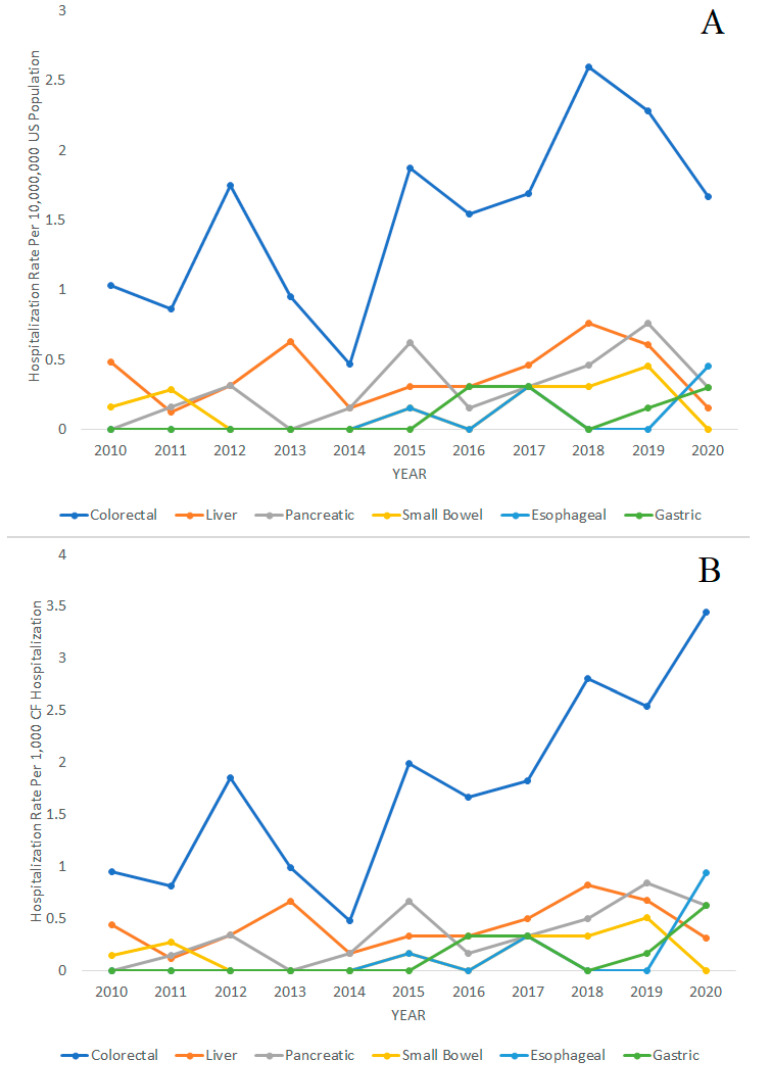
Hospitalization rates of colorectal, liver, pancreatic, small bowel, esophageal, and gastric cancer over time in patients with cystic fibrosis (CF), 2010–2020. (**A**) Hospitalization rates per 10,000,000 people in the general United States population. (**B**) Hospitalization rates per 1000 CF hospitalizations.

The trends in CF hospitalizations for gastrointestinal cancers over time from 2010 to 2020 are displayed in Figure 2. The figures display hospitalization rates with the general United States population as the denominator in Figure 2A and with CF hospitalizations as the denominator in Figure 2B. The following Poisson regression analysis for trends used the general United States population as the denominator. We found that the hospitalization rates for CF in general were decreasing from 2010 to 2020 (IRR = 0.96; 95% CI = 0.96–0.96; *p* < 0.001). However, the hospitalization rates of colorectal cancer (IRR = 1.09; 95% CI = 1.03–1.16; *p* = 0.005), pancreatic cancer (IRR = 1.17; 95% CI = 1.02–1.34; *p* = 0.023), gastric cancer (IRR = 1.41; 95% CI = 1.13–1.77; *p* = 0.003), and esophageal cancer (IRR = 1.50; 95% CI = 1.06–2.14; *p* = 0.023) in patients with CF were increasing during the same period. Liver cancer (IRR = 1.03; 95% CI = 0.92–1.16; *p* = 0.610) and small bowel cancer (IRR = 1.09; 95% CI = 0.89–1.34; *p* = 0.397) in CF hospitalizations did not significantly increase or decrease during the 2010–2020 period. 

For comparison, we also performed the Poisson regression analysis with total CF hospitalizations as the denominator and found the following results. The hospitalization rates of colorectal cancer (IRR = 1.14; 95% CI = 1.07–1.22; *p* < 0.001), pancreatic cancer (IRR = 1.23; 95% CI = 1.06–1.43; *p* = 0.006), gastric cancer (IRR = 1.53; 95% CI = 1.17–2.01; *p* = 0.002), and esophageal cancer (IRR = 1.66; 95% CI = 1.07–2.56; *p* = 0.023) in patients with CF were significantly increasing during the same period. Hospitalizations rates of liver cancer (IRR = 1.08; 95% CI = 0.95–1.22; *p* = 0.251) and small bowel cancer (IRR = 1.14; 95% CI = 0.92–1.43; *p* = 0.232) did not significantly increase or decrease during the 2010–2020 period.

We further explored the hospitalization rates of colorectal cancer and liver cancer, the two most common gastrointestinal cancers in our cohort, among CF patients with and without solid organ transplantation. The hospitalization rate trends for colorectal cancer and liver cancer in our CF cohort from 2010 to 2020 are displayed in Supplementary Figure S2 and Supplementary Figure S3, respectively. We conducted the Poisson regression analysis for trends using the general United States population as the denominator and found an upward trend in the hospitalization rate of colorectal cancer in the CF cohort that had solid organ transplantation (IRR = 1.23; 95% CI = 1.05–1.45; *p* = 0.012). The trends in colorectal cancer in the CF cohort without solid organ transplantation (IRR = 1.06; 95% CI = 0.99–1.13; *p* = 0.076), and liver cancer in the CF cohort with solid organ transplantation (IRR = 1.08; 95% CI = 0.81–1.43; *p* = 0.612) and without solid organ transplantation (IRR = 1.02; 95% CI = 0.90–1.15; *p* = 0.796), were not found to significantly increase or decrease over time. However, we also conducted Poisson regression analysis with total CF hospitalizations as the denominator and found the following results. There was a significant upward trend in the hospitalization rate of colorectal cancer in the CF cohort with solid organ transplantation (IRR = 1.31; 95% CI = 1.09–1.57; *p* = 0.004) and without solid organ transplantation (IRR = 1.11; 95% CI = 1.03–1.19; *p* = 0.012). The hospitalization rates of liver cancer in the CF cohort with solid organ transplantation (IRR = 1.13; 95% CI = 0.83–1.53; *p* = 0.446) and without solid organ transplantation (IRR = 1.06; 95% CI = 0.93–1.21; *p* = 0.390) did not significantly increase or decrease over time.

We compared the rates of gastrointestinal cancers between CF-associated hospitalizations and randomized age-matched, sex-matched, and race-matched asthma-associated hospitalization controls. The demographic and geographic characteristics of our CF cohort and our control group are displayed in Table 2. Colorectal cancer was significantly more common in the CF cohort compared to the asthma cohort, 0.167% versus 0.128% (*p* = 0.037). Rates of liver cancer, pancreatic cancer, small bowel cancer, gastric cancer, and esophageal cancer were similar between the CF cohort and asthma control group (*p* > 0.05) (Table 3).

The median age for hospitalization with gastrointestinal cancers within the randomized age-matched, sex-matched, and race-matched asthma-associated hospitalization controls were 46 (IQR: 35–56) years for colorectal cancer, 54 (IQR: 24–64) years for liver cancer, 54 (IQR: 42–69) years for pancreatic cancer, 42 (IQR: 33–60) years for small bowel cancer, 41 (IQR: 32–59) years for gastric cancer, and 52 (IQR: 43–62) years for esophageal cancer. These ages were statistically similar to those in the CF cohort for the respective gastrointestinal cancers (*p* > 0.05), with the exception of gastric cancer, in which the median age was significantly greater in the CF cohort (*p* < 0.001).

## 4. Discussion

This study is one of the largest of its kind in the United States and provides important insights into the rates of gastrointestinal cancers in the CF population and how they have evolved over time. We have also described the demographic and geographic characteristics of the CF patients who developed these gastrointestinal cancers. Additionally, we are the first to describe in detail the age distribution of CF-associated hospitalizations in the United States (Figure 1), providing valuable information on the burden of CF. 

Overall, we found that among the gastrointestinal cancers, colorectal cancers were increasing over time in the CF population relative to both the United States population and CF hospitalizations. The increased risk of gastrointestinal cancers in the CF population has been consistently reported in the literature, and among those, colorectal cancers were the most common [4,5,10]. The increasing hospitalization rates of colorectal cancer over time are likely due to the continually improving expected age of survival in the CF population, with implementation of the relatively recently approved triple combination drug elexacaftor, tezacaftor, and ivacaftor likely to play a major role [3,11]. There is also a trend in the general population in the United States of increasing incidence of colorectal cancer diagnoses in young adults, specifically those 49 years of age or younger, over the past several years, which could be contributing to the increasing rates of colorectal cancer in the CF population over time as well [12]. Although there has been an increase in the rate of early screening colonoscopies in patients with CF in recent years, which would also allow for therapeutic removal of any adenomas found during colonoscopy, the continued increase in colorectal cancer rate may suggest that these early screening interventions may not be practiced universally or they simply have not yet had time to show results. Additionally, we also found pancreatic cancer, gastric cancer, and esophageal cancer hospitalizations to be increasing over time in the CF population relative to both the United States population and CF hospitalizations. Our analysis comparing the rates of pancreatic cancer, gastric cancer, and esophageal cancer in the CF hospitalizations and controls did not find them to be significantly higher, suggesting that the development of those cancers in our CF cohort may not be influenced by the intrinsic factor(s) of CF disease itself as encountered with the rates of colorectal cancer. A case–control study by McWilliams et al. reported that pathogenic mutations in CFTR could be associated with a modest increase in risk for pancreatic cancer, but the study was limited by a lack of control for age and a small sample size [13]. Furthermore, as screening for pancreatic cancer is still not a routine procedure and screening methods remain poor, we believe the primary driver of the increased hospitalization rates of pancreatic cancer to be the increased survival age of the CF population. 

Liver cancers are not routinely screened for in the general population, but may be indirectly screened for in CF patients as CF-associated liver diseases are increasingly being recognized and monitoring liver function has been standard practice [14]. This routine liver monitoring may explain the notably young age of the CF cohort with liver cancer compared to the other gastrointestinal cancers in our study. We believe viral hepatitis was not likely to be contributing to the early development of liver cancer in the group considering the very low frequencies of hepatitis B and hepatitis C in this cohort. Nevertheless, further research is needed to more clearly explain this interesting finding. 

In our study, the increased risk of colorectal cancer in the CF cohort compared to controls is evident. Colorectal cancer is routinely screened for in the general population, starting at 45 years of age with colonoscopy, and this has been increasingly implemented in the CF population at younger ages. In our study, we found the median age of colorectal cancer-associated hospitalization to be 46 years of age, suggesting that routine colorectal screening in the CF population should begin at least 10 years prior, at approximately 36 years of age or younger, as the adenoma–carcinoma sequence is typically believed to take more than 10 years to complete in sporadic cancers [15]. However, due to the overall younger average age of the CF population compared to the general population, there could be biases in directly comparing the median ages of colorectal cancer. As such, we also directly compared the CF cohort to our asthma controls which had the same age distribution due to age matching; here, we found that the median ages of colorectal cancer hospitalizations were similar between the two groups. In 2018, Hadjiliadis et al. reported consensus recommendations for colorectal cancer screening in CF patients. The group recommended colonoscopy as the preferred screening method with initiation of screening at age 40 years, 5-year re-screening, and 3-year surveillance intervals [16]. 

The mortality rate of 28.6% in CF hospitalizations with gastric cancer was the highest in our cohort; however, considering the overall small number of CF hospitalizations with gastric cancer, there may be bias in this mortality rate. The next highest mortality rate in our cohort was pancreatic cancer, which is consistent with the very high rates of mortality of pancreatic cancer in the general population. The 5-year survival rate of pancreatic cancer within the general population after diagnosis, including all stages, is only 12% [17]. 

The mechanism by which CFTR dysfunction contributes to increased gastrointestinal cancer risk is not well understood. However, it is thought to be caused by a combination of multiple mechanisms. Studies have previously shown that individuals with CF have chronic increased gastrointestinal epithelial cell turnover that begins in infancy and early childhood, which would increase the risk of deleterious mutations in those cells [18,19]. The dysfunction of the CFTR protein also results in increased viscosity of the luminal secretions which impairs mucociliary clearance, leading to mucosal obstruction and inflammation with neutrophilic predominance [20]. This chronic inflammation causes direct damage to the epithelial cells and contributes to bacterial dysbiosis in the gut [21,22], which is further worsened by the frequent use of antibiotics in this population. There is increasing evidence of the contribution of the gut microbiota to the development of colorectal cancer in the general population [23]. It has also been theorized that the relatively high radiation burden of the CF population, from frequent X-rays and CT scans, may contribute to the increased risk of gastrointestinal cancers [7]. 

Overall, the rates of gastrointestinal cancer within the CF cohort compared to our matched controls were largely similar, except for colorectal cancer, which occurred at higher rates in the CF cohort. However, this difference in colorectal cancer rates is actually quite modest, being only around 30% higher than in the matched control. This sharply contrasts with many prior studies including a systematic review and meta-analysis by Yamada et al. which reported significantly increased risks of small bowel cancer, colon cancer, biliary tract cancer, and pancreatic cancer, with pooled standardized incidence ratios of 18.94, 10.91, 17.87, and 6.18, respectively, compared to the general population [7]. That study and several other studies on the topic are limited by the lack of age-matched controls and rely on indirect methods of age adjustment and may not even include race/ethnicity adjustment. The systematic review and meta-analysis by Yamada et al. also relied on multiple studies, each of which had very few cases of gastrointestinal cancer, potentially causing a bias in extrapolation. In our study, we only found a statistical difference in the rates of colorectal cancer, but nowhere near the degree of difference described by Yamada et al. From the results of our study, we believe that the early colorectal cancer screening proposed by Hadjiliadis et al. could be justified [16], but at this time, there is insufficient evidence to justify increased screening for other types of gastrointestinal cancers.

There are some limitations to this study. As the National Inpatient Sample is a database of hospitalizations and does not identify unique individuals, the same individual could be hospitalized multiple times in the same year without any method to track or exclude those individuals. As such, we could not compare our hospitalization rates of gastrointestinal cancers to incidence data in the general United States population and had to use asthma hospitalizations as a control. As CF patients are prone to having multiple hospitalizations during their lifetime, there may be a detection bias for CF hospitalizations compared to other hospitalizations even our asthma hospitalization controls. There is potential for miscoding in the database; we assumed that any miscoded diagnoses would be distributed randomly between the cohorts, without statistical impact. Another limitation is the difficulty of finding an appropriate control group for the CF cohort, which is a very demographically unique population, being quite young and being predominately non-Hispanic White in race/ethnicity. Furthermore, this cohort is also younger than the average general population in the United States, making comparisons to the general population difficult. In order to address these issues, we selected another cohort within the National Inpatient Sample with a respiratory disease that was known to not increase the risk of cancer, a cohort of asthma hospitalizations, to become our control group. Furthermore, we decided to create a control group with similar age, sex, and race/ethnicity distributions to the CF cohort by matching them in a ratio of 2 to 1 in all three of those demographics.

## 5. Conclusions

Overall, gastrointestinal cancers are a major cancer burden in the CF population. In particular, colorectal cancers are the most common among gastrointestinal cancers and occur at higher rates compared to age-matched controls. Colorectal, pancreatic, gastric, and esophageal cancers are also increasing over time within the CF population. Mortality was highest in gastric and pancreatic cancers in the CF population. The increase in gastrointestinal cancer risk in the CF population may be more modest than previously reported. Further research exploring the factors leading to cancer within the CF population would be beneficial.

## Figures and Tables

**Table 1:** Demographic and geographic data among gastrointestinal cancers in cystic fibrosis (CF)-associated hospitalizations, 2010–2020.

	Colorectal Cancer	Liver Cancer	Pancreatic Cancer	Small Bowel Cancer	Gastric Cancer	Esophageal Cancer
**Number of Hospitalizations, N**	539	139	105	54	35	30
Age, Year, Median (IQR)	46 (37–57)	39 (21–54)	53 (40–60)	53 (37–62)	65 (54–79)	59 (35–75)
Sex						
Male, N (%)	264 (49.0)	84 (60.4)	60 (57.1)	35 (64.8)	20 (57.1)	20 (66.7)
Female, N (%)	275 (51.0)	55 (39.6)	45 (42.9)	19 (35.2)	15 (42.9)	≤10
Race/Ethnicity						
Non-Hispanic White, N (%)	446 (82.7)	119 (85.6)	100 (95.2)	49 (90.7)	25 (71.4)	25 (83.3)
Non-Hispanic Black, N (%)	19 (3.5)	≤10	0 (0.0)	0 (0.0)	0 (0.0)	0 (0.0)
Hispanic, N (%)	25 (4.6)	15 (10.8)	0 (0.0)	0 (0.0)	≤10	0 (0.0)
Asian or Pacific Islander, N (%)	≤10	0 (0.0)	0 (0.0)	0 (0.0)	0 (0.0)	0 (0.0)
Native American, N (%)	≤10	0 (0.0)	0 (0.0)	0 (0.0)	0 (0.0)	0 (0.0)
Other/Unknown, N (%)	39 (7.2)	0 (0.0)	≤10	≤10	≤10	≤10
Region of Hospital						
Northeast, N (%)	112 (20.8)	29 (20.9)	15 (14.3)	≤10	15 (42.9)	≤10
Midwest, N (%)	138 (25.6)	30 (21.6)	35 (33.3)	24 (44.4)	≤10	≤10
South, N (%)	208 (38.6)	60 (43.2)	50 (47.6)	≤10	≤10	≤10
West, N (%)	80 (14.8)	20 (14.4)	≤10	≤10	≤10	≤10
Type of Hospital						
Rural, N (%)	≤10	0 (0.0)	≤10	0 (0.0)	0 (0.0)	≤10
Urban Non-Teaching, N (%)	58 (10.8)	≤10	15 (14.3)	≤10	≤10	≤10
Urban Teaching, N (%)	471 (87.4)	134 (96.4)	80 (76.2)	44 (81.5)	25 (71.4)	20 (66.7)

**Table 2:** Demographic and geographic characteristics of cystic fibrosis (CF)-associated hospitalizations and randomized age-matched, sex-matched, and race-matched asthma-associated hospitalization controls, 2010–2020.

	Cystic Fibrosis Cases	Matched Controls
**Number of Hospitalizations, N**	323,594	567,442
Age, Year, Median (IQR)	22 (16–32)	23 (16–32)
Sex		
Male, N (%)	147,964 (45.7)	256,630 (45.2)
Female, N (%)	175,630 (54.3)	310,812 (54.8)
Race/Ethnicity		
Non-Hispanic White, N (%)	252,236 (77.9)	470,487 (82.9)
Non-Hispanic Black, N (%)	14,449 (4.5)	28,723 (5.1)
Hispanic, N (%)	26,096 (8.1)	50,628 (8.9)
Asian or Pacific Islander, N (%)	1400 (0.4)	2702 (0.5)
Native American, N (%)	1309 (0.4)	2533 (0.4)
Other/Unknown, N (%)	28,104 (8.7)	12,369 (2.2)
Region of Hospital		
Northeast, N (%)	52,649 (16.3)	125,497 (22.1)
Midwest, N (%)	81,604 (25.2)	133,161 (23.5)
South, N (%)	127,856 (39.5)	197,492 (34.8)
West, N (%)	61,487 (19.0)	111,293 (19.6)
Type of Hospital		
Rural, N (%)	8266 (2.6)	48,197 (8.5)
Urban Non-Teaching, N (%)	21,647 (6.7)	137,293 (24.2)
Urban Teaching, N (%)	291,848 (90.2)	380,036 (67.0)

**Table 3:** Rates of gastrointestinal cancers in cystic fibrosis (CF)-associated hospitalizations compared to randomized age-matched, sex-matched, and race-matched asthma-associated hospitalization controls.

	Cystic Fibrosis	Matched Controls	*p*-Value
**Total Hospitalizations**	323,594	567,442	
Colorectal Cancer, N (%)	539 (0.167)	725 (0.128)	**0.037**
Liver Cancer, N (%)	139 (0.043)	234 (0.041)	0.846
Pancreatic Cancer, N (%)	105 (0.032)	212 (0.037)	0.603
Small Bowel Cancer, N (%)	54 (0.017)	49 (0.009)	0.125
Gastric Cancer, N (%)	35 (0.011)	114 (0.020)	0.146
Esophageal Cancer, N (%)	30 (0.009)	74 (0.013)	0.480

Bolded *p*-values are statistically significant, <0.05.

## Data Availability

The data presented in this study are available on request from the corresponding author. The data are not publicly available due to the privacy policy of the Healthcare Cost and Utilization Project (HCUP).

## Supplementary Materials

The following supporting information can be downloaded at https://www.mdpi.com/article/10.3390/diagnostics14181999/s1: Supplementary Table S1: International Classification of Diseases, Ninth Revision (ICD-9) and International Classification of Diseases, Tenth Revision (ICD-10) diagnosis codes for variables used in study. Supplementary Figure S1: Hospitalization rates of cystic fibrosis (CF)-associated hospitalizations over time per 10,000,000 people in the general United States population, 2010–2020. Supplementary Figure S2: Hospitalization rates of colorectal cancer over time in patients with cystic fibrosis (CF) with and without solid organ transplantation, 2010–2020. (A) Hospitalization rates per 10,000,000 people in the general United States population. (B) Hospitalization rates per 1000 CF hospitalizations. Supplementary Figure S3: Hospitalization rates of liver cancer over time in patients with cystic fibrosis (CF) with and without solid organ transplantation, 2010–2020. (A) Hospitalization rates per 10,000,000 people in the general United States population. (B) Hospitalization rates per 1000 CF hospitalizations.

## References

[1] Scotet V., Gutierrez H., Farrell P.M. (2020). Newborn Screening for CF across the Globe-Where Is It Worthwhile?. Int. J. Neonatal Screen..

[2] Southern K.W., Munck A., Pollitt R., Travert G., Zanolla L., Dankert-Roelse J., Castellani C., ECFS CF Neonatal Screening Working Group (2007). A survey of newborn screening for cystic fibrosis in Europe. J. Cyst. Fibros..

[3] Marshall B., Faro A., Brown W. (2021). Cystic Fibrosis Foundation Patient Registry: 2020 Annual Data Report. https://www.cff.org/sites/default/files/2021-11/Patient-Registry-Annual-Data-Report.pdf.

[4] Neglia J.P., FitzSimmons S.C., Maisonneuve P., Schöni M.H., Schöni-Affolter F., Corey M., Lowenfels A.B. (1995). The Risk of Cancer among Patients with Cystic Fibrosis. N. Engl. J. Med..

[5] Maisonneuve P., Marshall B.C., Knapp E.A., Lowenfels A.B. (2013). Cancer Risk in Cystic Fibrosis: A 20-Year Nationwide Study From the United States. JNCI J. Natl. Cancer Inst..

[6] Vajdic C.M., van Leeuwen M.T. (2009). Cancer incidence and risk factors after solid organ transplantation. Int. J. Cancer.

[7] Yamada A., Komaki Y., Komaki F., Micic D., Zullow S., Sakuraba A. (2018). Risk of gastrointestinal cancers in patients with cystic fibrosis: A systematic review and meta-analysis. Lancet Oncol..

[8] HCUP-US NIS Overview. https://hcup-us.ahrq.gov/nisoverview.jsp.

[9] Bureau U.C. Population and Housing Unit Estimates. https://www.census.gov/popest.

[10] Maisonneuve P., FitzSimmons S.C., Neglia J.P., Campbell P.W., Lowenfels A.B. (2003). Cancer Risk in Nontransplanted and Transplanted Cystic Fibrosis Patients: A 10-Year Study. JNCI J. Natl. Cancer Inst..

[11] McBennett K.A., Davis P.B., Konstan M.W. (2022). Increasing life expectancy in cystic fibrosis: Advances and challenges. Pediatr. Pulmonol..

[12] American Cancer Society (2020). Colorectal Cancer Facts & Figures 2020–2022.

[13] McWilliams R.R., Petersen G.M., Rabe K.G., Holtegaard L.M., Lynch P.J., Bishop M.D., Highsmith W.E. (2010). Cystic fibrosis transmembrane conductance regulator (CFTR) gene mutations and risk for pancreatic adenocarcinoma. Cancer.

[14] Debray D., Kelly D., Houwen R., Strandvik B., Colombo C. (2011). Best practice guidance for the diagnosis and management of cystic fibrosis-associated liver disease. J. Cyst. Fibros..

[15] Rex D.K., Boland C.R., Dominitz J.A., Giardiello F.M., Johnson D.A., Kaltenbach T., Levin T.R., Lieberman D., Robertson D.J. (2017). Colorectal Cancer Screening: Recommendations for Physicians and Patients From the U.S. Multi-Society Task Force on Colorectal Cancer. Gastroenterology.

[16] Hadjiliadis D., Khoruts A., Zauber A.G., Hempstead S.E., Maisonneuve P., Lowenfels A.B., Braid A.L., Cullina J., Daggett A., Fink A. (2018). Cystic Fibrosis Colorectal Cancer Screening Consensus Recommendations. Gastroenterology.

[17] (2023). Cancer Facts & Figures 2023.

[18] Pang T., Leach S.T., Katz T., Jaffe A., Day A.S., Ooi C.Y. (2015). Elevated fecal M2-pyruvate kinase in children with cystic fibrosis: A clue to the increased risk of intestinal malignancy in adulthood?. J. Gastroenterol. Hepatol..

[19] Garg M., Leach S.T., Pang T., Needham B., Coffey M.J., Katz T., Strachan R., Widger J., Field P., Belessis Y. (2018). Age-related levels of fecal M2-pyruvate kinase in children with cystic fibrosis and healthy children 0 to 10years old. J. Cyst. Fibros..

[20] Elborn J.S. (2016). Cystic fibrosis. Lancet.

[21] Arthur J.C., Perez-Chanona E., Mühlbauer M., Tomkovich S., Uronis J.M., Fan T.-J., Campbell B.J., Abujamel T., Dogan B., Rogers A.B. (2012). Intestinal inflammation targets cancer-inducing activity of the microbiota. Science.

[22] Werlin S.L., Benuri-Silbiger I., Kerem E., Adler S.N., Goldin E., Zimmerman J., Malka N., Cohen L., Armoni S., Yatzkan-Israelit Y. (2010). Evidence of intestinal inflammation in patients with cystic fibrosis. J. Pediatr. Gastroenterol. Nutr..

[23] Kim J., Lee H.K. (2022). Potential Role of the Gut Microbiome In Colorectal Cancer Progression. Front. Immunol..

