# Supplementary material for: Gastrointestinal Cancers in Hospitalized Patients with Cystic Fibrosis: A Nationwide Study, 2010–2020
Source: Diagnostics (Basel). 2024 Sep 10;14(18):1999. doi: 10.3390/diagnostics14181999 (PMC11431327; doi:10.3390/diagnostics14181999)

Supplementary Table S1. International Classification of Diseases, Ninth Revision (ICD-9) and International Classification of Diseases, Tenth Revision (ICD-10) diagnosis codes for variables used in study.

Diagnosis	ICD-9 Codes	ICD-10 Codes
Cystic Fibrosis	270.0*	E84*
Asthma	493*	J45*
Colorectal Cancer	153*, 154*	C18*, C19, C20
Liver/Intrahepatic Bile Duct Cancer[1]	155*	C22*
Pancreas Cancer	157*	C25*
Small Bowel Cancer	152*	C17*
Gastric Cancer	151*	C16*
Esophageal Cancer	150*	C15*
Solid Organ Transplantation History	V42.0, V42.1*, V42.6*, V42.7*, V42.83, 996.81, 996.82, 996.83, 996.84, 996.86, 996.87	Z94.0*, Z94.1*, Z94.2*, Z94.3*, Z94.4*, Z94.82*, Z94.83*, T86.1*, T86.2*, T86.3*, T86.4*, T86.81*, T86.85*
Hepatitis B	070.2*, 070.3*, 070.42, 070.52	B16*, B17.0, B18.0, B18.1, B19.1*
Hepatitis C	070.41, 070.44, 070.51, 070.54, 070.7*	B17.1*, B18.2, B19.2*

1. Includes hepatocellular carcinoma and intrahepatic cholangiocarcinoma.

Supplementary Figure S1. Hospitalization rates of cystic fibrosis (CF)-associated hospitalizations over time per 10,000,000 people in the general United States population, 2010-2020.

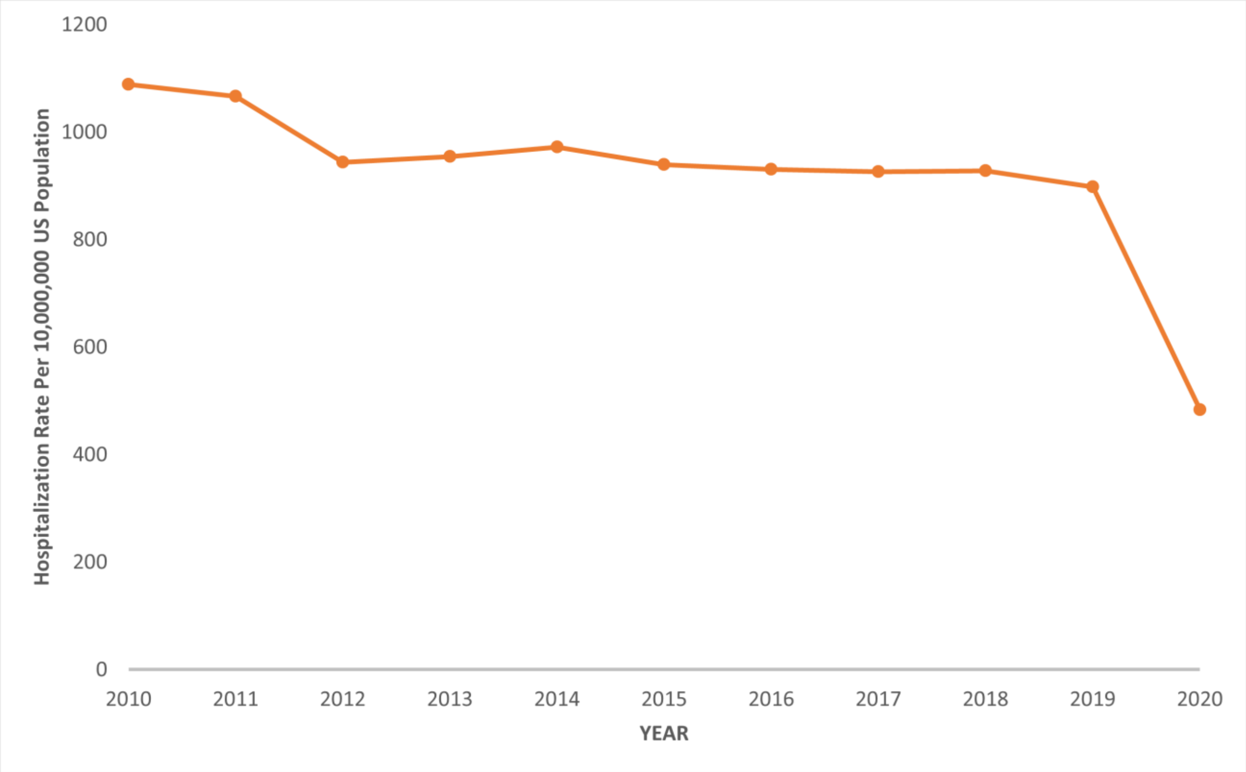

Supplementary Figure S2. Hospitalization rates of colorectal cancer over time in patients with cystic fibrosis (CF) with and without solid organ transplantation, 2010-2020. (A) Hospitalization rates per 10,000,000 people in the general United States population. (B) Hospitalization rates per 1,000 CF hospitalizations.

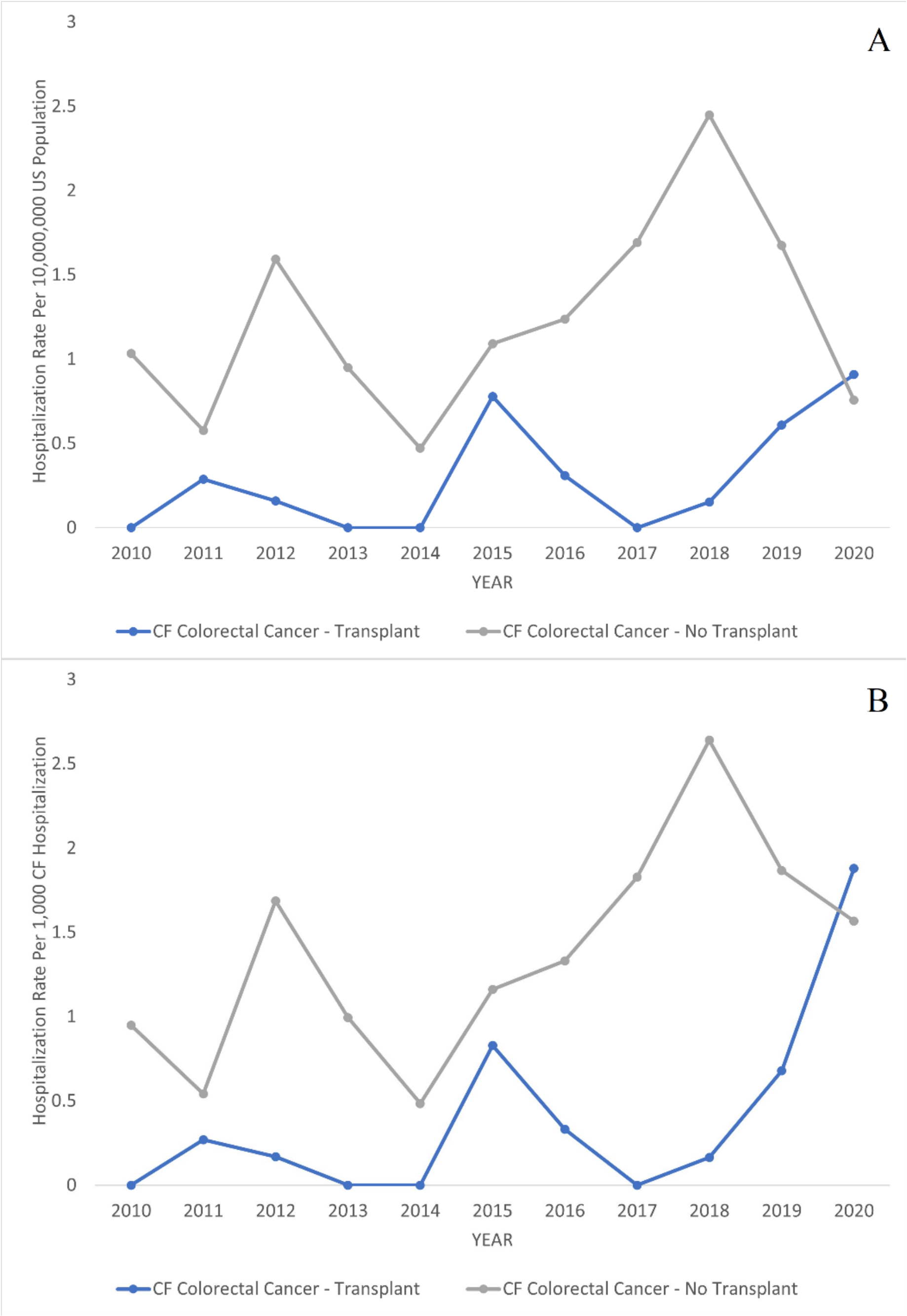

Supplementary Figure S3. Hospitalization rates of liver cancer over time in patients with cystic fibrosis (CF) with and without solid organ transplantation, 2010-2020. (A) Hospitalization rates per 10,000,000 people in the general United States population. (B) Hospitalization rates per 1,000 CF hospitalizations.

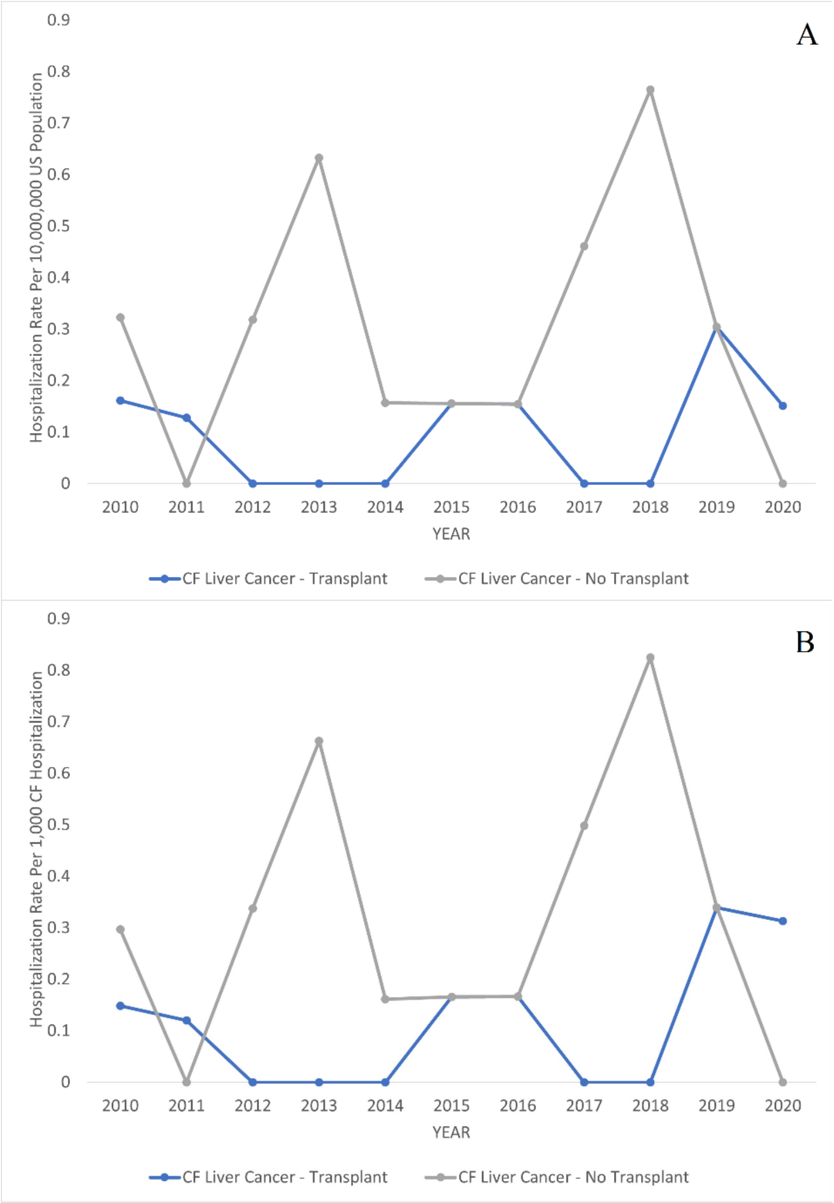

Supplement: Supplementary file 1 [file diagnostics-14-01999-s001.zip › diagnostics-3163188-supplementary.pdf]
